
JOURNAL INFORMATION
==============================
NLM Title Abbreviation: Atlanta Med Surg J (1884)
NLM Journal ID: 101251623

Atlanta Medical and Surgical Journal (1884)

Publisher: Biomedical Journal Digitization Project

ARTICLE INFORMATION
==============================
PMCID: PMC9199294
Article version: 1
Subjects: Book Reviews, Pamphlets, Exchanges

Literary Notes

Print publication date: 1898 Dec
Volume: 15
Volume Series: New Ser
Issue: 10
First page: 705
Last page: 705
License: This work is free of known copyright restrictions. For more information, please see PMC Back Issue Digitization.
License URL: https://creativecommons.org/publicdomain/mark/1.0/

==============================

Modern Treatment of Tuberculosis. By Charles Denison, A.M., M.D., Denver, Col.

Bulletin of the Johns Hopkins Hospital, for September and October.

This issue is filled with excellent articles from the Anatomical Library of the University. It is a pleasure to see such eminently scientific work being done in an American university.

A Rapid Treatment of Chancroid and Ulcerative Syphilitic Lesions. By A. H. Ohmann-Dumesnil, A.M., M.D., of St. Louis.

---

### *LITERARY NOTES.*

---

FOUR HUNDRED PRETTY HOMES AND GARDENS.—How general the use of photography is coming to be adopted by the modern magazine as a means of illustration is shown in the announcement of *The Ladies' Home Journal* that it is about to publish six new, distinct series of articles which will include not less than 400 photographs. The idea of the magazine is to present one hundred of the prettiest country homes in America to encourage artistic architecture; one hundred of the prettiest gardens, to encourage taste in floriculture; seventy churches decorated for festal occasions of all kinds, such as weddings, Christmas and Easter services, etc.; some forty of the prettiest girls' rooms in this country; twenty-five floral porches and vine-clad houses; and the story of the native wild flowers in America, told in seventy-five photographs. Over 8,000 photographers, in every part of the country, were employed by the magazine to get these pictures, and several thousands of dollars were paid in prize awards for the best photographs. The choice was made out of over 10,000 photographs received by the magazine.

---

LONGFELLOW TO BE ILLUSTRATED.—Last year Charles Dana Gibson illustrated "The People of Dickens" for *The Ladies' Home Journal.* The pictures were so successful that this year, and during next year, W. L. Taylor, the New England artist who has made such rapid strides in his art, will illustrate "The People of Longfellow"—also for *The Ladies' Home Journal.* The poems selected are "The Psalm of Life," "Hiawatha," "Evangeline," "The Courtship of Miles Standish," "The Children's Hour," "The Village Blacksmith," and others.
